# From complication to litigation: The importance of non-technical skills in the management of complications *

**Published:** 2020-08-05

**Authors:** ESGE special interest group ‘quality , safety and legal aspects’ working group, AA Watrelot, V Tanos, G Grimbizis, E Saridogan, R Campo, A Wattiez

**Affiliations:** Hospital Natecia, Lyon, France;; Nicosia University Medical School and Aretaeio Hospital, Nicosia, Cyprus;; Aristotle University, Thessaloniki, Greece;; University College London Hospital, London, United Kingdom;; Life Center, Leuven, Belgium;; Latifa Hospital, Dubai, United Arab Emirates.

**Keywords:** complications, gynaecological surgery, litigation, malpractice, medicolegal

## Abstract

Complications do occur in daily clinical life and can sometimes lead to litigation, which adversely affect the entire health care system, leading to a loss of confidence in medical providers, an increase in defensive medical practice and high professional indemnity insurance costs. Some complications are inevitable but can be minimised by completing a structured training programme. The likelihood of litigation can be reduced when adequate and clear information is given to the patient preoperatively. Non-technical skills are essential in complication management and crucial if confronted with litigation. Checklists and documentation of medication and surgical steps should be routine in all surgeries. Awareness of the complexity of the planned operation, theatre set-up and equipment are important in preventing complications. Mental preparation of surgeons is of the utmost importance in order to be able to confront any problem. When complications occur, remaining calm, calling for assistance, effective team leadership and harmony in the team are important in managing the situation. Good and effective communication with the patient and relatives, offering explanations, apologies and timely intervention without delays reduce the risk of litigation and strengthen any defence in court.

## Introduction

Complications occur during and after surgical treatment and some of them may be inevitable. Occasionally, patients and relatives consider the treatment which led to the complication as negligent or malpractice and may initiate litigation. The law regulating medical practice varies significantly between countries, but as a general rule, litigation follows when it is perceived that the medical practitioner has failed to provide competent care. Unfortunately, litigation adversely affects the entire health care system, leading to a lack of confidence in medical providers, an increase in defensive medical practice and very high professional indemnity or insurance costs. Litigation can be minimised by prevention, early diagnosis and proper management of complications.

When medicolegal cases are under scrutiny, experts take the experience of individual surgeons into account. The court also examines the evidence and then judges if there is negligence. Therefore, lack of training or certification in a specific surgical field, such as certification in minimally invasive surgery, could influence the decision when a surgeon eventually faces litigation. Demonstration of experience by way of record of training or certification may eliminate any dispute of the surgeon’s credibility (Tanos et al., 2016).

The likelihood of litigation can be minimised when adequate and clear information is passed to the patient prior to an operation. Not all complications are considered negligent and their characterisation as such, is highly dependent on the expert’s opinion. The experts and lawyers need to see convincing evidence that the patient has been given adequate information to make a decision prior to surgery. This may be achieved by adopting a patient centred approach.

## A patient centred approach

Websites and social media enable patients to have easy access to information on their health condition. However, they may not be able to assess the accuracy of information and may be unable to identify or distinguish their symptoms and link them with a specific condition in a comprehensive way (Hirsch et al., 2020). The surgeon has a key role in explaining this volume of information clearly and giving the patient a complete picture of the illness, satisfying their needs and expectations regarding the planned surgery.

It is recommended to provide written information consisting of:

- a detailed explanation of planned surgery (many societies or patient support organisations provide detailed information on all major types of operations)
- expected/intended benefits of the operation
- possible complications (e.g. in case of laparoscopy for deep endometriosis the risk of bowel injury/ denervation),
- a brief explanation of the options of different techniques, and
- therapeutic alternatives including expectant management.

This approach will help the physician secure the confidence of the patient and may facilitate reaching agreements on difficult management options such as re-intervention or 2-step surgery. A structured and ‘patient centred approach’ to delivering information on the planned operation, obtaining consent and documenting the details of consultation enable the surgeon to focus on the medical aspects of possible complications with less fear of a complaint or litigation. It is important to develop a good patient-doctor relationship, starting from the first consultation and maintaining it. Disagreements between the patient and their doctors are more likely to result in litigation when complications occur.

When a complication occurs, it is essential to be open and honest about what happened. It is also important to listen to the patient and hear their concerns. Relatives and family should also be informed, with the patient’s permission. In France and some other countries, the patient gives the name of a person of trust who may receive information. Usually, these are close relatives, like a parent or husband who are seeking information and have the right to know, unless the patient specifically requests otherwise. Respecting the patient’s right to privacy and dignity is of the utmost importance and overrides the rights of others.

## Complication management

Since most laparoscopic complications are uncommon and permanent injuries are even less common, few surgeons are familiar and prepared to deal with these unpleasant processes. However, non-technical skills are of the utmost importance, besides the technical skills necessary to manage a complication (Mishra et al., 2008).

The aviation industry was the first among all professions to demonstrate that safety increases tremendously when the training of pilots included human factor risk management approach, so called Crew Resource Management (CRM). Unfortunately, this was after the catastrophic accident of Tenerife in 1987, where more than 560 passengers lost their lives, due to bad communication in the cockpit. Most of the principles of CRM may be adapted to minimally invasive surgery (MIS) (particularly in laparoscopic surgery) to improve patient safety.

## Importance of ‘non-technical skills’ in the management of a complication

The purpose of this document is to provide an overview of the best approach required to minimise litigation and its consequences following complications in MIS. The ‘non-technical skills’ do not substitute the technical skills, but they are a necessary counterpart and complement one another (Arora et al., 2011).

**Awareness** that any adverse event may occur from the time the patient enters the operating room (OR) until the moment they leave is crucial (Lam et al., 2009). Complications are not limited to complex laparoscopic operations, even simple interventions carry these risks.

During laparoscopy, the first trocar entry is a blind procedure. Approximately 50% of laparoscopic complications have been reported to occur during the first entry, demonstrating that consciousness and alertness are required at the very beginning of surgery. When a big vessel injury is evident, it is expected to be treated immediately. However, in case of difficult adhesiolysis, ongoing awareness would be required to watch for subtle injuries such as small bleeding or minor bowel trauma and meticulous inspection of the abdominal cavity is needed. Awareness should also be combined with reason and sense. For example, an abrupt decrease of the blood pressure should be considered as a result of a haemorrhage until proven otherwise. If a surgeon wrongly believes that is due to anaphylactic shock it may lead to an irrecoverable and fatal outcome.

**Self-control.** In case of an acute complication, the surgeon’s behaviour is critical. The unfortunate episode is always unexpected and sudden. It is important for a surgeon to control their temper. Extremes of reactions such as shaking and shouting or, on the contrary, total apathy lead to a loss of valuable time and the endangers patient’s safety further. A common scenario is what it is called the ‘tunnel effect’ where the surgeon focuses on a central point and becomes totally oblivious to the surrounding events (lack of situation awareness). This should be avoided. The key to success is to be mentally prepared for acute complications and remain calm. A surgeon’s inability to control their stress frequently results in the transmission of their frustration to the whole operating room (OR) team, causing confusion and further unnecessary delays.

In all situations the surgeon must remain in control. A typical example is a case of massive haemorrhage during laparoscopy. Successful management will depend on the reaction of the surgeon to this stressful situation. The response will be observed and interpreted by the team. A correct attitude will be respected and highly appreciated by the team.

The surgeon is the team leader and should act accordingly. He is responsible for maintaining cohesion of the group, motivating the team and listening to each member. They should assume the role of the leader and make it clear that the members of the OR team should listen to their requests, follow their instructions and perform in serious, fast and efficient manner. A team in which everyone has an equal say might sound like the correct democratic attitude. However, this approach has proven to have limitations, especially in case of complications where there is no place for lengthy discussions. In these situations, effective leadership is crucial. In addition, it is important to develop a no-blame culture, which gives confidence to the team to perform their best without any fear of penalty in case of error.

**Post-operative complication management** is often an unpleasant situation, especially when a re- intervention is imperative. It is usually difficult for the patient to accept and may be even more difficult for the surgeon to decide for the re-intervention. The feeling of failure should be avoided since it may delay the decision to re-operate. After an appropriate preoperative counselling and consent process, the patient will have been aware of the potential risks and will be willing to accept. The time lag between the occurrence of the complication and the re- intervention will be scrutinised by the experts in case of litigation. The surgeon should perform a close follow up of the patient especially during the first post-operative week. In addition, it is preferable to perform an explorative laparoscopy for no pathology than to do it at a later stage with eventual irrepairable damages. Seeking assistance from a colleague, such as a specialist for the organ involved in the complication is also very important. Besides medical help, their presence will be considered by experts as a sensible decision in case of future medico-legal action. When a repeat operation is performed, ideally all required procedures should be carried out to treat the complication completely during the session. The risk of litigation increases tremendously depending on the number of additional procedures after a complication. A third operation substantially increases the risk of litigation.

## Prevention of litigation is often possible when a complication occurs

Informing the patients and their relatives when complications are detected and providing an explanation should be executed with empathy, and in a clear and accurate way. It is important to debrief the OR team immediately after the complication to ensure that everybody understands what happened so that thoughts and views are aligned. Provision of different versions of events given by doctors and/or nurses increases the likelihood of litigation. When the patient is transferred to an intensive care unit in another hospital, briefing the team is even more difficult and can sometimes be impossible.

Doctor-patient communication is very crucial when complications arise. The surgeon should have the skills to break bad news. In addition to showing empathy, it is important to reassure the patient that they are receiving the appropriate treatment by competent professionals for the emerging new problem. It is also important to reassure them that the doctor or the team will be monitoring the progress of the condition and, providing updates to them or selected relatives. The surgeon or their team needs to make themselves available for the patient and the family throughout the recovery period.

It is critical for the surgeon and/or their team to take time and visit the patient regularly postoperatively and, provide updates and explanation on progress. Additionally, detailed records must be kept so that there is a traceability of events. This is critical and will be very helpful in case of litigation.

Surgeons may be hesitant to report their complications even after successful management. This apprehension may be due to the risk of complications being associated with malpractice, regardless of their validity or the fear that it may lead to questions on the surgeon’s competence. Reporting complications is indeed a difficult task, since the difference between complications and malpractice may be a fine line. For example, in a patient with symptomatic deep endometriosis, adhesiolysis resulting in bowel perforation and delayed diagnosis is likely to be considered malpractice, when the surgeon fails to diagnose the perforation in a timely manner. Therefore, any complication may be interpreted as negligent by individuals who are not familiar with the principles of medicolegal practice.

## The difference between complication and malpractice and the importance of pre-operative information to patients

As explained earlier, the line between malpractice and complication is a fine line and is not always very clear. It depends on several parameters and in a court of law, the judge will conclude if there is malpractice or not, with help from experts. Examples of what is considered malpractice are found in daily practice. The injury of a nearby organ/structure, for example injury to ureter in its normal anatomic position during a simple ovarian cystectomy, is likely to be considered malpractice. Vascular injuries during entry should not occur, if MIS principles are respected. Therefore, they are more difficult to defend, unless the surgeon clearly demonstrates they have followed all reasonable safety measures designed to reduce risk of this type of injury. In contrast, when the anatomy is distorted, as frequently found in endometriosis, it is very clear that these injuries would not be considered malpractice. A medical report written immediately after the operation is therefore critical to be able to defend the case if there is a subsequent medico- legal claim (Ngo et al., 2016) Delays in recognizing a complication or delays in early intervention when it is obviously necessary, a lack of accurate history and risk factors prior to the procedure and a lack of required details in the operation report are also examples of poor practice which make defence difficult or impossible.

It is important to keep appropriate records to demonstrate that the patient has been given adequate information about the operation, its material risks and alternative options. There may be differences in different countries as to how this documentation should be kept. It is good practice to give/send a summary of the consultation to the patient. In some countries, there is an expectation that this information should be written by the surgeon and counter-signed by the patient. This would help in case of litigation, so the patient cannot claim ignorance, or insufficient information and an absence of the written information provided. The consent form is more formal and could be identical in general aspects for every surgical procedure. Doctors have a legal duty to obtain a competent patient’s consent prior to any treatment, apart from exceptional circumstances. This form explains clearly that the patient has received specific information, has understood the explanations given and has no further queries or questions. A time gap between the issue of information and the signing of the two documents (the consent form and information form), and the surgery is accepted as good practice by international standards. To ensure patient understanding, a ‘quick exit quiz’ can be placed at the end of the consent process by asking simple questions about complications, alternative options including expectant management. If there are misunderstood items or aspects that need more clarification, it may be wise to arrange another formal consultation with the patient prior to the day of surgery.

In many European countries the doctor is responsible for proving that correct and adequate information is given and only signed consent and information forms are admissible evidence in court. If the doctor does not have control of or free access to hospital records, or there is a possibility that hospital records may be lost, it is imperative that doctors keep their own records, and do not rely solely on the hospital records. In many cases, the failure of hospital patient records systems or, incomplete or missing information puts the onus of proof on the doctor rather than the hospital.

## Association of the OR set up and technical support in the prevention of complication manifestation

Quality of equipment, credibility, accountability and dedication of the team are involved in litigation processes.

Surgeons must ensure that the equipment in the OR is of good quality and that there is sufficient technical support and maintenance. It is of utmost importance that the camera and monitor are of high quality to support viewing with good clarity. Instrumentation insulation and frequent testing throughout the year, depending on the number of operations, is imperative. Electrosurgical burns secondary to direct coupling may develop from faulty insulation of a monopolar or bipolar tool, so these should be checked regularly and taken out of circulation when detected. Instruments for an emergency laparotomy should be easily accessible and available at all times in case conversion to open surgery is needed. Similarly, vascular clamps should be stored in such a place that every member of the team can find them without delay. Details of specialists from other disciplines such as vascular surgery should be easily available in the OR. It is good practice to have standard operating procedures for these types of emergencies. A check list is mandatory. A proposed specific check list for laparoscopy recommended by the ESGE is available on https://esge.org/wp-content/uploads/2020/06/ESGE-Checklist-Laparoscopy.pdf and should be carefully followed (Appendix). Most important is to have instruments and equipment in good condition, checked for good insulation and functionality for any laparoscopic operation for complex as well as for routine procedures. The OR team should ensure the correct patient identification, the type of registered procedure, availability of appropriate equipment and OR set-up. Antibiotic and other medication prophylaxis that may be required should be available and the time of drug administration must be documented. Any anticipated difficulties or problems during the operation need to be highlighted.

## Steps to be followed when complications occur during MIS

### The best example: major haemorrhage during MIS:

#### - The first steps:

Once serious unexpected bleeding is identified, the initial step is to stop the bleeding by applying pressure with a clamp or any other instrument in the abdominal cavity and attempts to coagulate blindly or coagulation of major vessels should be avoided. Then a decision should be made whether or not to convert to laparotomy, depending on the injured vessel. Bleeding control and any conversion decision should be accomplished within seconds, requiring the surgeon to maintain self-control, take the appropriate decision and respond rapidly. There should not be a debate on conversion to laparotomy if the surgeon judges that, either due to their insufficient surgical experience or due to team/OR circumstances. In these circumstances it is better to manage the complication by open surgery. This is why the risk of conversion should be always mentioned to the patient pre-operatively. Regular simulation and drills for the team is of great help building up team alertness and readiness to such eventualities (Rochlen et al., 2019).

#### - Call for Help:

To call a colleague for assistance is one of the first things to do in case of a serious adverse event. Even if a surgeon specialised in the type of complication involved is not available, any colleague would be useful to reduce the stress, to have a more objective look on the situation and to do whatever is surgically necessary, whilst waiting for more appropriate medical help such as a vascular surgeon to arrive. It is important to remember that it is not necessarily a weakness to call for help and, on the contrary, one would be expected to act this way. Once prepared, then immediate action considered by experts is the most sensible thing to do which will take care of any future medico-legal action.

#### - Team attitude:

It is well known from the aviation world that in any complex activity, like MIS, on average 3 to 5 mistakes occur per hour. 80% are corrected by the expert and 20% by the team, hence teamwork is important (Mishra et al., 2008).

## Should a surgeon offer apology to the patient after a complication?

It is good practice to apologise when things go wrong. In fact, in some countries such as England there is a duty of candour which requires that healthcare professionals must tell a patient when something has gone wrong, apologise, offer a remedy or support and provide full explanation. This does not mean they accept negligence, on the contrary it reduces litigation, particularly in case of minor injuries (Wojziescak et al., 2006, Dahan et al., 2017). If there is a significant possibility that the surgeon is at fault, an independent expert review may be necessary. This process has been followed in some states in America and is starting in some European countries like France. They propose mediation by an independent reviewer with the help of patient support organisations followed by a quick compensation in case of malpractice. It seems that this approach is very promising, and the first USA results show that when no fault is the outcome of investigation, less than 20% of patients will finally sue the doctor and less than 10% will obtain financial compensation. In the United Kingdom, hospitals carry out their own investigation, initially by an internal investigator, and sometimes by an independent external investigator, when serious incidents occur.

## Conclusion

Complications during surgery do occur. Good knowledge of anatomy, disease physiology and pathogenesis as well as the technical skills certification are of paramount importance in minimising the number and severity of these adverse events. When a complication occurs, litigation depends on a number of factors which include patient information, a good relationship between the patient and their doctors and proficiency of non-technical skills. Complication management is critical not only in optimising the medical outcome but also in preventing medico-legal consequences. Surgeons are not usually well prepared at medical school for these incidents and professional organisations/societies such as the ESGE may have a role in initiating educational programmes for the necessary non-technical skills.

## Abbreviations

Malpractice: careless, wrong or illegal behaviour while in a professional job.
Negligence: the failure to give somebody/something enough care or attention.
Litigation: the process of making or defending a claim in court
